# Combination of transcriptomic and metabolomic analyses reveals a JAZ repressor in the jasmonate signaling pathway of *Salvia miltiorrhiza*

**DOI:** 10.1038/srep14048

**Published:** 2015-09-21

**Authors:** Qian Ge, Yuan Zhang, Wen-Ping Hua, Yu-Cui Wu, Xin-Xin Jin, Shuang-Hong Song, Zhe-Zhi Wang

**Affiliations:** 1 Key Laboratory of the Ministry of Education for Medicinal Resources and Natural Pharmaceutical Chemistry, National Engineering Laboratory for Resource Development of Endangered Crude Drugs in Northwest of China, College of Life Sciences, Shaanxi Normal University, Xi’an, China; 2 Co-Innovation Center for Qinba regions’ sustainable development, College of Life Sciences, Shaanxi Normal University, Xi’an, China

## Abstract

Jasmonates (JAs) are plant-specific key signaling molecules that respond to various stimuli and are involved in the synthesis of secondary metabolites. However, little is known about the JA signal pathway, especially in economically significant medicinal plants. To determine the functions of novel genes that participate in the JA-mediated accumulation of secondary metabolites, we examined the metabolomic and transcriptomic signatures from *Salvia miltiorrhiza*. For the metabolome, 35 representative metabolites showing significant changes in rates of accumulation were extracted and identified. We also screened out 2131 differentially expressed unigenes, of which 30 were involeved in the phenolic secondary metabolic pathway, while 25 were in the JA biosynthesis and signal pathways. Among several MeJA-induced novel genes, *SmJAZ8* was selected for detailed functional analysis. Transgenic plants over-expressing *SmJAZ8* exhibited a JA-insensitive phenotype, suggesting that the gene is a transcriptional regulator in the JA signal pathway of *S. miltiorrhiza*. Furthermore, this transgenic tool revealed that JAZ genes have novel function in the constitutive accumulation of secondary metabolites. Based on these findings, we propose that the combined strategy of transcriptomic and metabolomic analyses is valuable for efficient discovery of novel genes in plants.

*Salvia miltiorrhiza* is a well-known traditional Chinese medicine, and considered as a model medicinal plant^1^,^2^. In Asia, its dry roots have been widely used for hundreds of years in the prevention and treatment of menstrual disorders, cardiovascular diseases, acute ischemic stroke diseases, and inflammation^3^. The active ingredients of *S. miltiorrhiza* are divided into two main groups: lipid-soluble tanshinones and water-soluble phenolic acids. Both are secondary metabolites, levels of which are not only controlled by plant genotype but also enhanced by various biotic and abiotic stresses. The abiotic elicitor methyl jasmonate (MeJA) dramatically increases the biosynthesis of active ingredients in those species^4^,^5^,^6^. Therefore, this phenomenon might be exploited as a potential strategy for improving the production of valuable secondary metabolites by regulating the biosynthesis of jasmonates^7^. To data, numerous metabolites have been isolated and identified from *S. miltiorrhiza*^8^. In view of the economic value of its active ingredients, studies on the tanshinone^9^ and phenolic acid biosynthetic pathways^10^ are drawing considerable attention in recent years. Although several genes involved in metabolite biosynthesis are known to be induced by JA treatment, only a few have been identified through classical genetic screening.

Jasmonic acid (JA) and its MeJA derivative, collectively called jasmonates, are signaling molecules with regulatory functions in wound responses and some developmental processes^11^,^12^. The well-established JA biosynthesis pathway is shown in 13. Signal transduction begins with the recognition of JA by the Skp1/Cullin/F-box (SCF) complex through the ubiquitin-proteasome system. The Jasmonate Zim Domain (JAZ) proteins are targets of the SCF^COI1^ complex, and are subsequently subjected to proteasomal degradation in order to release positively acting transcription factors (TFs), such as MYC2 and MYB^14^,^15^,^16^. Exogenous application of MeJA results in strong activation of a large set of target genes and biochemical substances. Of interest to us are those involved in the JA-mediated biosynthesis of secondary compounds. We have obtained the proof of concept for this JA-dependent induction from studies of constitutive accumulation of caffeoylputrescine in tomato (*Solanum lycopersicum*)^17^, nicotine biosynthesis in tobacco (*Nicotiana tabacum*)^18^, and JA-induced anthocyanin formation^19^. In *S. miltiorrhiza*, Luo *et al.*^20^ reported 7 candidate cytochrome P450s which may be involved in the tanshinone and phenolic acid biosynthesis by investigating MeJA-induced gene expression profiles. However, JA-induced signal pathway and its connection with the downstream biosynthesis of active ingredients are still need to further investigate.

Integrated analyses of the metabolomes and transcriptomes of *Arabidopsis*^21^ and tomato^22^ have already led to the discovery of several new genes with special functions. This approach was also utilized to characterize the inducible nature of tanshinone production in hairy root cultures of *S*. *miltiorrhiza*^23^. These reports strengthened our confidence that such approaches maybe effective in comprehensive analyses of JA-induced gene expression and metabolite accumulations in *S. miltiorrhiza*. Furthermore, after previously combining the EST database with Solexa sequencing, we have obtained 57,754 unigenes, which comprises the entire collection of general genetic data currently available for that species^24^,^25^. Our laboratory has also developed mass spectrometry-based technologies for targeted and non-targeted metabolomic analysis^26^. By implementing these background and novel methods, we hope to provide robust evidence upon which to evaluate any relationship between the JA pathway and metabolic biosynthesis in *S. miltiorrhiza*. As part of our first step, we report here our comprehensive analysis of the impact that MeJA has on *S. miltiorrhiza* along with a phenotype description, transcriptome data, and results from the metabolomic analysis. In this study, we also selected a candidate gene induced by MeJA that encodes JAZ protein. This gene, *SmJAZ8*, was identified as a repressor in the JA signaling pathway. Its abundance of transcript was linked with extraordinary suppression of the regulation of JA-dependent responses in *S. miltiorrhiza*. Transgenic analysis also suggested its potential role in JAZ-regulated secondary metabolism, based on significantly increased levels of salvianolic acid B (Sal B) measured in the transgenics. These findings will help in further establishing an efficient strategy as “phenotype-transcriptome-metabolome” for discovering novel genes in those economically significant medicinal plants.

## Results

### “Phenotype-metabolome-transcriptome” analysis of *Salvia miltiorrhiza* under MeJA treatment

**Figure 1:**
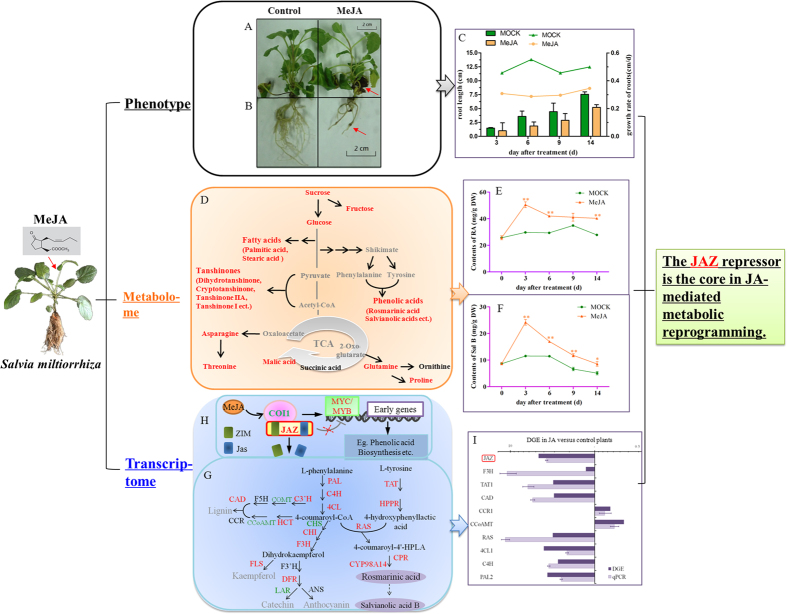
Response by *Salvia miltiorrhiza* to MeJA. Phenotype of shoots (**A**) and roots (**B**) upon treatment. (**C**) Effects of MeJA on root growth relative to MOCK control. (**D**) Map of major metabolic pathways relative to secondary metabolaism of *S. miltiorrhiz.* Red font indicates that metabolite level was changed by more than 1.5-fold (*p* < 0.05) when compared with untreated control; black, no significant changes; gray, undetectable. Time course of MeJA effects on contents of rosmarinic acid, RA (*E*) and salvianolic acid, SalB (*F*) in MOCK seedlings under 0.2% ethanol treatment and in MeJA seedlings under 0.1 mM treatment. *significant at p < 0.05; **highly significant at p < 0.01. (*G*) proposed biosynthetic pathway for phenolics, beginning with core phenylpropanoid and tyrosine pathways, and leading to 3 major branch pathways: phenolic acid, flavonoids, and lignin (up-regulated genes in red; down-regulated genes in green). ANS, anthocyanin synthase; CAD, cinnamyl alcohol dehydrogenase; CCR, cinnamoyl-CoA reductase; CCoAMT, caffeoyl-CoA O-methyltransferase; CHI, chalcone isomerase; CHS, chalcone synthase; COMT, caffeic acid O-methyltransferase; CYP, cytochrome P450enzymes; CPR, cytochrome P450 reductase; C3′H, coumarate 3′-hydroxylase; C4H, cinnamate 4-hydroxylase; DFR, dihydroflavonol 4-reductase; F3H, flavanone 3-hydroxylase; F3′H, flavonoid 3′-hydroxylase; F5H, ferulate 5-hydroxylase; FLS, flavonol synthase; HCT, hydroxyl cinnamoyl transferase; HPPR, hydroxyl phenylpyruvate reductase; LAR, leucocy anidin reductase; PAL, phenylalanine ammonia lyase; RAS, rosmarinic acid synthase; TAT, tyrosine aminotransferase; 4CL, hydroxycinnamate-CoA ligase. (**H**) MeJA signal pathway. (**I**), qRT-PCR verification of DGE tag data for *JAZ* (Unigene36977); *F3H* (Unigene54426); *TAT1* (Unigene56449); *CAD* (Unigene37246); *CCR1* (Unigene53802); *CCoAMT* (Unigene39895); *RAS* (Unigene55331); *4CL1* (Unigene54261); *C4H* (Unigene53935) and *PAL2* (Unigene52119). All data are means of 3 replicates, with error bars indicating SD.

Plants exposed to MeJA also displayed massive, spontaneous necrotic lesions on leaves nearest the base of the plant. This necrosis eventually engulfed the entire surface of older leaves so that they became dark and died by Day 14 (Fig. 1A). It has been known for a long time that root growth is inhibited by JA or its methyl ester^27^. To investigate the effects of MeJA in *S. miltiorrhiza*, we initially cultured plants for 3 d in an MS basal solid medium before exposing them to either 100 μM MeJA or a mock solution. A scan showed that roots harvested from JA-treated seedlings were shorter and had a significantly slower growth rate when compared with the phenotype of roots from control plants (Fig. 1B). On Day 14 of these observations, the treated plants exhibited a moderate increment in average root diameters but significantly fewer (by 90%) lateral roots (Fig. 1C, Table S1).

We suspected that, to a large extent, this influence on the morphological phenotype depended upon modulations in primary metabolism. Therefore, we performed GC/MS-based profiling for primary metabolites as well as phenolic- and tanshinone-targeted analyses by LC/MS.

As expected, we identified 16 primary metabolites as differential contributors (Table S2). These included amino acids (threonine, proline, asparagine, and glutamine), carbohydrates (D-fructose, D-ribose, glucose oxime, D-mannitol, mannitol, galactose oxime, sucrose, and lactose), organic acids (malic acid and butanoic acid), and fatty acids (palmitic acid and stearic acid). Those compounds strongly influenced plant growth. In addition, the levels of secondary metabolites were determined with LC-MS (Table S3), and a total of 19 compounds were differentially accumulated between the two groups.

In all, we extracted 132 and 125 independent analytes that represented putative metabolites obtained from the LC/MS and GC/MS data sets, respectively. After peak alignments, both sets were assessed by the unsupervised PCA and PLS-DA method. Differences between the two groups were distinct (Figure S2). When combined with our finding from the score and loading plots (Figure S3), these results suggested that both primary and secondary metabolite contents had been altered by MeJA stimulus (Fig. 1D).

It is widely believed that rosmarinic acid (RA) and salvianolic acid B (Sal B) are the active phenolic ingredients in *S. miltiorrhiza*^8^,^28^. Therefore, we selected RA and Sal B as the representative compounds for further investigation. Their accumulations in response to MeJA were monitored over 0, 3, 6, 9, and 14 days, respectively. Duplicate analyses were conducted via HPLC to develop a time course of reactions (Fig. 1E,F). Levels of RA increased up to Day 3, peaking at 29.68 ± 0.09 mg g^−1^ DW when compared with the corresponding control. The accumulation of Sal B was also highest on Day 3 before declining. Previous research has shown that accumulations of RA and Sal B in *S. miltiorrhiza* are highest when exposed to 100 μM MeJA for 6 d and that those levels are correlated with increments in gene expression^6^. Hence, we concluded that these enhanced accumulations were probably a result of increased levels of transcripts for genes involved in those metabolic pathways.

Using the Solexa/Illumina DGE system, we found a total of 3,843,001 and 3,787,000 tags in our control and MeJA libraries. After low-quality tags were filtered out, 3,819,543 and 3,763,892 “clean” tags remained, respectively. These were mapped to the previously reported reference library^24^ for bioinformatics purposes. Approximately 48% of all clean tags could be mapped to >25% of the predicted *S. miltiorrhiza* genes, i.e., 25,251 and 23,298 genes for the control and MeJA libraries, respectively (Table S4). Overall, 0.82% of these genes were significantly up-regulated by more than 5-fold, whereas the expression of 1.05% of all genes was decreased in the MeJA library (FDR ≤ 0.001 and |log2Ratio|≥1; Figures S4, S5). We used PCA to compare the transcript profiles between MOCK and MeJA-treated plants. The differences in those profiles were similar to the contrasts found in metabolite profiles between the two libraries (Figure S2, S3). The KEGG pathway was analyzed to improve our understanding of DEG biological functions and to identify genes potentially involved in crosstalk between the JA signaling pathway and important biochemical pathways. Overall, we found that our DEGs participated in 116 pathways, with 24 being significantly enriched in DEGs at *p* < 0.05 and an FDR value < 0.01 (Table S5). Those functions included the metabolism of phenylpropanoids and alkaloids, as well as the biosynthesis of hormones, phenylpropanoids, and flavonoids. Based on previous experiments conducted in our laboratory, we chose to focus on genes in the pathways for phenolic acids and mapped them with KEGG pathway annotations to predict candidate genes important for JA-induced metabolism. Phenolic compounds are produced from phenylalanine and tyrosine through a series of hydroxylation, methylation, and dehydration reactions (Fig. 1G). Expression of 8 genes in the phenylpropanoid pathway increased more than 2-fold, especially *PAL*, *4CL*, *RAS*, *CYP98A14*, and *CPR* (Table S6, Fig. 1G). This discovery was consistent with an earlier report of JA-induced expression in *Larix gmelinii*^29^. We detected three genes in the tyrosine pathway, where expression was enhanced by 3.10-fold to 5.57-fold over the control. Other genes were involved in the phenylpropanoid pathway to regulate the production of lignin and flavonoids^30^,^31^. We noted that the abundances of several transcripts in those two pathways were elevated after plants were exposed to MeJA whereas expression of nine genes was reduced. We also found it of interest that some of the unigenes annotated in same gene shown different expression patterns, and vice versa. For example, expression of *Unigene2612* was down-regulated while that of *56687* was up-regulated simultaneously, even though both were annotated by flavanone 3′-hydroxylase (F3′H).

In addition to analyzing the abundance of genes for enzymes in the biosynthesis pathway for phenolics, we determined whether upstream changes in the JA signal pathway are responsible for the extraordinary accumulation of metabolites in MeJA-treated plants. As described previously, JA biosynthesis is regulated by a positive feedback loop^32^. Of those genes, most had higher transcripts under MeJA treatment (Fig. 1H). In particular, *LOX* expression was increased by up to 133.00-fold when compared with the control.

*Jasmonoyl isoleucine conjugate synthase* (*JAR*) catalyzes the final step in the formation of the bioactive JA compound^27^. That gene was the only one in the JA biosynthesis pathway that was significantly suppressed. Such a cycle of expression may have contributed to the fine-tuning of JA signaling. Similar to previous reports^33^, we found that the transcripts of five unigenes annotated as *JAZ* were up to 98.12-fold more abundant in MeJA-treated plants (Table S6). Due to the core role of JAZ protein in the JA-mediated metabolic reprogramming, we thus focused on those ‘pop’ genes and analyzed their functions in details.

Eight up-regulated and two down-regulated genes were randomly selected as representatives within different pathways. Pair-wise comparisons of the normalized abundance of these 10 genes were made using results from our DGE and qRT-PCR analyses. These revealed similar patterns of induction by MeJA (Fig. 1I), which confirmed the reliability of our transcriptome data.

### Identification of novel *S. miltiorrhiza JAZ* genes

Conserved domains for ZIM and Jas were utilized to screen sequence fragments from our transcriptome database^24^ so that we could identify putative *JAZ*s from *S. miltiorrhiza*. Based on Blast results, we designed primers flanking coding regions that resembled other known *JAZ*s. Using leaf- and/or root-derived cDNAs, we cloned and sequenced PCR-amplified fragments, thereby yielding four distinct genes named *SmJAZ1*, *-2*, *-3*, and *-8* (corresponding GenBank Accession Numbers JQ936590, KC864779, KC864780, and JQ936591).

**Figure 2:**
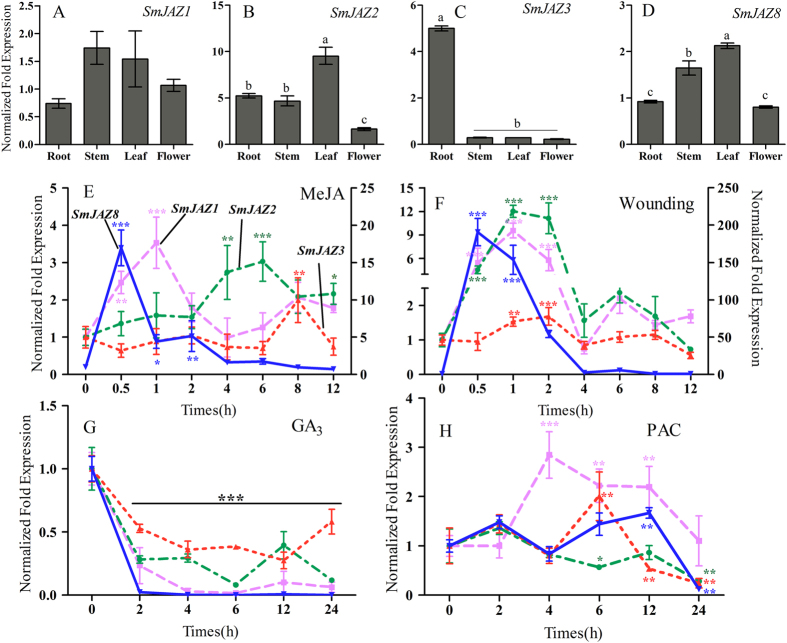
Expression patterns of individual *JAZ* genes in different tissues and under abiotic stresses. (**A**–**D**) Transcript abundances for *SmJAZ1* (**A**), *SmJAZ2* (**B**), *SmJAZ3* (**C**), and *SmJAZ8* (**D**) in root, stem, leaf, and flower. (**E**–**H**) Expression following treatment with 5 mM MeJA (**E**), mechanical wounding (**F**), 150 μM GA (**G**), and 150 μM PAC (**H**). Accumulations of *SmJAZ* transcripts were determined by qRT-PCR (n = 3). *β-Actin* was used as internal control. Fold-changes indicate expression relative to negative control, which was set to 1. Significant differences were determined at each time point compared with negative control, by Student’s *t*-tests (*P < 0.05, **P < 0.01, ***P < 0.001).

The expression patterns of *SmJAZ*s were studied with qRT-PCR as well as their response to stress. As our control, expression was monitored in untreated leaves to understand systemic induction. Whereas *SmJAZ1*, *-2*, and *-8* were mainly expressed in leaf and stem tissues, only *SmJAZ3* was more highly expressed in the roots (Fig. 2). Similar to previous reports^33^, all *SmJAZ*s showed strong and early induction by MeJA or wounding. GA treatment led to immediate down-regulation while PAC, an inhibitor of GA, caused *SmJAZ*s to be moderately up-regulated. These patterns were similar to those found with other species, which directly confirmed the universal characteristics of *JAZ* genes and suggested that their functions are conserved in hormone and defense pathways^34^. The response to wounding or MeJA was quite different between *SmJAZ8* and the other *SmJAZ*s, with transcripts of the former being more than 100-fold (wounding) and 17-fold (MeJA) higher at 0.5 h after elicitation (see scales along y-axes in Fig. 2). This suggested that, compared with its lack of detection under control conditions, *SmJAZ8* has special biological functions and is inducible only by certain stress stimuli.

A detailed structure analysis of *SmJAZ8* showed that it has three exons and two introns. Sequence alignments showed that, like other JAZ family members, SmJAZ8 contains the ZIM and Jas domains. Phylogenetic analysis of the full-length proteins from *S*. *miltiorrhiza* and members in the *Arabidopsis* JAZ family^33^ (AtJAZ1 to -12) showed that *SmJAZ* genes could be clustered with others in Subgroup III because it was most similar to AtJAZ7 and AtJAZ8 (Figure S6)^5^.

### Overexpression of *SmJAZ8* and phenotype analysis of jasmonate-responsiveness

To characterize the functioning of *SmJAZ8* in detail, we generated transgenic plants that over-expressed *SmJAZ8* (Figure S7). Our qRT-PCR analyses of expression in PCR-positive transgenics showed that *SmJAZ8* transcripts were accumulated by more than 2000-fold over the controls. Four lines exhibiting efficient overexpression were selected for functional analysis. However, the transgenic sterile plants were no difference from the controls in vegetative growth and development under normal conditions.

**Figure 3:**
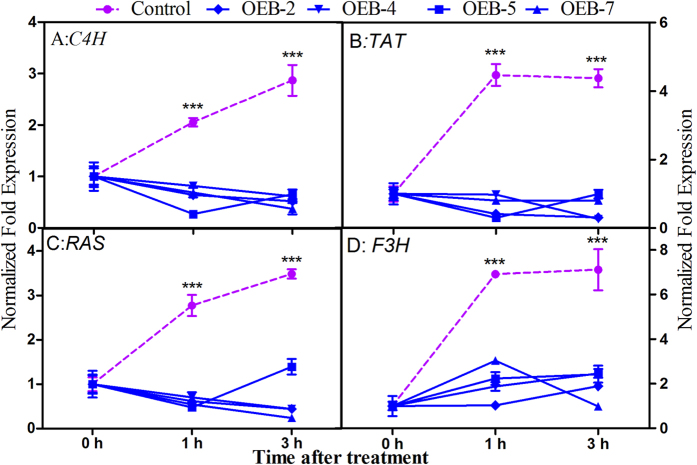
Patterns of expression by JA-responsive genes in OEB lines under MeJA treatment. Mean values and SDs were obtained from 3 technical and 3 biological replicates.

To evaluate the role of *SmJAZ8* in the JA signaling pathway, we monitored the expression of inducible genes *SmC4H*, *SmTAT*, *SmRAS*, and *SmF3H* (based on DGE results above) in OEB and control plants following MeJA exposure. Their transcripts in the control lines were substantially induced early while none of those genes was induced in any transgenic line within the first 3 h of treatment (Fig. 3). The OEB plants seemed to be JA-hyposensitive presumably because of their high level of *SmJAZ8* expression.

The inhibition of root growth by exogenous JA has long been used to screen for JA-insensitive mutants such as the coi1 mutant^35^. Jasmonate is also reportedly involved in the formation of lateral roots^36^. Because SmJAZ8 likely represses MeJA-induced genes, its overexpression should result in weak constitutive physiological responses, e.g., reductions in root growth or development of lateral roots. When our three-day-old seedlings received exogenous MeJA, the root growth of MeJA-treated control group was obviously inhibited when compared with either untreated plants or treated OEB lines. Statistical analysis of those data also confirmed that this overexpression of *SmJAZ8* buffered the influence of MeJA on lateral root formation (Table 1).

In *S. miltiorrhiza*, the predominant active ingredients, RA and Sal B, are enriched by MeJA. To examine whether this is consistent with the transgenics, we used HPLC to measure their accumulations after 14 d of treatment. Whereas differences in Sal B contents did not differ noticeably between control and overexpressing plants (except for Line OEB-5), levels of RA were significantly lower in the OEB lines (Table 2). Namely, the JA-induced metabolite increments seemed to be reduced partially in transgenic plants.

### Levels of total phenolics and flavonoids, and antioxidant properties of plant extracts

**Figure 4:**
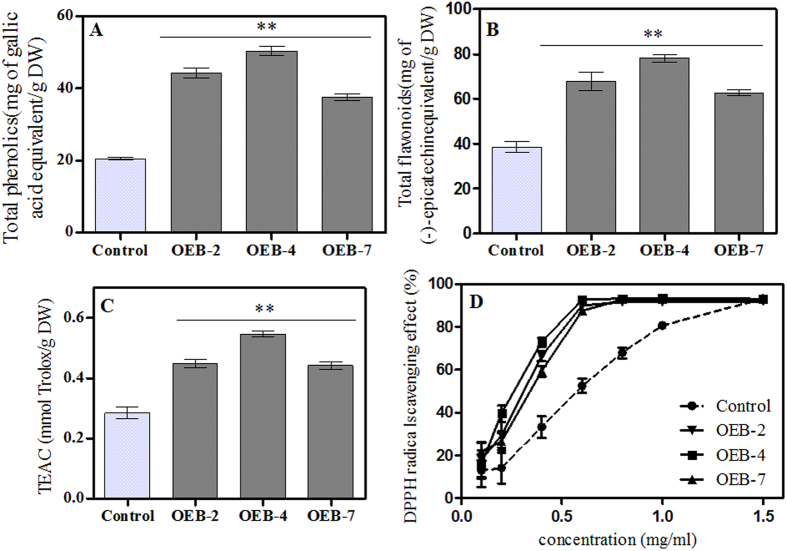
Changes in targeted secondary metabolites and antioxidant capacity between control and OEB lines. Levels of total phenolics (**A**) and flavonoids (**B**) from root extracts. (**C**) curve for DPPH free radical scavenging. (**D**) Trolox equivalent antioxidant capacity. Values followed by **within a column are significantly different from the control at *p* < 0.01.

Although *SmJAZ8* overexpression did not affect plant growth and development, the amounts of less-essential secondary metabolites may have been altered. Therefore, we performed global assays for phenolics and flavonoids. In contrast to our expectations, the levels of both were higher in OEB lines than in the controls. In particular, the strongest increments in constitutive levels were found in OEB-4, i.e., 2.45-fold higher for total phenolics and 1.75-fold higher for flavonoids (Fig. 4A,B).

Secondary metabolites, especially phenolic compounds, may have bioactive properties and be important contributors to antioxidant, antimicrobial, and anti-inflammatory activities^37^,^38^. Thus, the over-accumulation of flavonoids may enhance crop tolerances to both biotic and abiotic stresses^39^. Here, the antioxidant capacity of our transgenic plants was obviously enhanced (Fig. 4C,D). Calculations revealed that the EC_50_ value in OEB-4 was 0.237 mg mL^−1^, which was significantly higher than the 0.537 mg mL^−1^ measured for the control. In contrast, none of the samples showed activity as strong as V_C_. These results suggested that overexpressing plants had a higher level of secondary metabolites that was most likely responsible for their greater antioxidant activities.

### Variations in specific soluble phenolic acids

**Figure 5:**
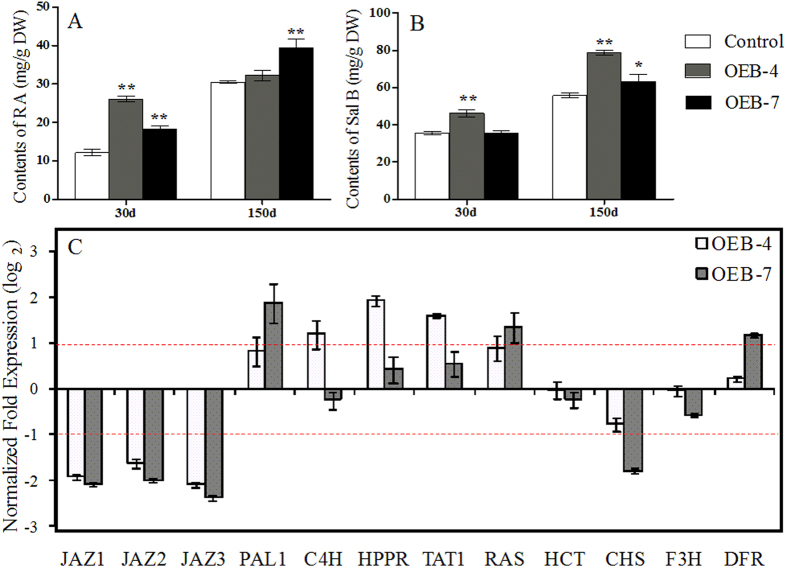
Specific changes in concentrations of RA (A) and Sal B (B) in roots from OEB and control lines, based on HPLC results. (**C**) verification of relative gene expression via qRT-PCR. Fold-changes reflect expression in OEB-4 and OEB-7 compared with control expression, for which values were set to 1 (not shown).

We performed HPLC analysis to study the levels of RA and Sal B, two major hydrophilic and active pharmaceutical ingredients in *S. miltiorrhiza.* We also investigated how their metabolism was modified in OEB plants. Concentrations were determined at 30 d (just after plants were transferred to the greenhouse) and again at 150 d. The Sal B concentration was highest (78.74 ± 1.48 mg g^−1^ DW) in OEB-4 roots sampled at 150 d, and was 1.41-fold higher then, as well as 1.30-fold higher on Day 30, than corresponding levels in the control (Fig. 5). Similarly, amounts of RA were significantly higher (by 1.52-fold to 2.15-fold) in one-month-old OEB lines. However, those levels began to decline after 150 d. Overall, the status of Sal B was more stable than that of RA, presumably due to that Sal B is the final product in the phenolic acid pathway.

To evaluate whether these substantially elevated accumulations of RA and Sal B in the transgenics were a result of greater gene expression relative to the control, we examined transcript levels for nine key structural genes in the phenolic biosynthesis pathway as well as three other *SmJAZ*s. When normalized to the control, those three *SmJAZ*s were shown to be down-regulated simultaneously by 19 to 32% (Fig. 5). Expression of *PAL*, *hydroxyl phenylpyruvate reductase* (*HPPR*), *TAT*, and *RAS*, which are required for RA synthesis, was up-regulated in OEB plants. We observed no distinct increases in the transcripts of *hydroxyl cinnamoyl transferase* (*HCT*), *chalcone synthase* (*CHS*), and *F3H*, all of which are involved in pathways for flavonoid and lignin biosynthesis. Transcripts of *dihydroflavonol 4-reductase* (*DFR*) were higher only in OEB-7. Those results were consistent with findings from our previous study^40^, in which Sal B concentrations were enhanced when gene expression increased in the biosynthesis pathway.

## Discussion

### The power of integrating transcriptome and metabolome data for functional genomics

In the process of discovering two glycosyltransferase genes putatively involved in anthocyanin synthesis, researchers recognized that an approach combining metabolomics and transcriptomics can be an efficient means for functional classification of genes^21^. To complete their examination of how enzyme genes and the pathways for flavonoid metabolism are modified, Keiko *et al.*^30^. identified several novel genes from a pool of candidates by coupling their detailed analysis of secondary metabolites with transcriptome coexpression analysis. Those reports provided strong evidence that integrating transcriptome and metabolome data can be applied to studies of roles by hypersensitive response-associated genes in tomato^41^. By combining metabolomic and transcriptomic analysis, terpenoid metabolism were demonstrated to display a biphasic response to elicitation^23^, and a potential role for the induced MeJA-responsive transcription factor was found. However, studies focused on the phenolic acid pathways are still lacking. Transcriptional profile of *S. miltiorrhiza* leaves in response to MeJA induction was detected by RNA-seq technology and the results shown a significant transcriptional complexity^20^. Subsequently, the same group described 29 genes related to phenolic acid bioynthesis from the *S. miltiorrhiza* genome^10^, and provided a rich gene pool for phenolic acid biosynthesis. The extensive functional evidences and their responses to JA induction are of great importance. Here, we used these methods to reveal the holistic changes associated with exogenous MeJA. Massive re-programming occurred in our JA-treated plants, and we found DEGs, most of which were up-regulated, that belonged to 116 pathways.

Crosstalk between the JA signal pathway and the secondary metabolism pathway is one of the best examples of cooperation among genes to fulfill their biological functions. Because MeJA treatment was linked with increased transcript abundance, it also led to a marked enrichment in levels of primary and secondary metabolites. For example, two active ingredients, RA and Sal B, were accumulated in concentrations much above normal levels. Such results can improve our understanding of the roles played by individual components in the JA response, and these findings help to explain how the functional genes examined here contribute to this metabolomic network.

### Conserved functioning of *SmJAZ8*, a novel JAZ gene in *S. miltiorrhiza*

The JAZ proteins are targets of the SCF^COI1^ complex; their degradation releases some positively acting TFs to initiate transcription of JA-responsive genes^42^. However, Thines *et al.*^15^ have produced transgenic lines in *Arabidopsis* with complementary DNAs that encode JAZ proteins under the CaMV *35S* promoter. None of those plants exhibit an obvious jasmonate-related phenotype. Other reports have indicated that only overexpression or a reduction in the transcript abundance of alternative splice variants of AtJAZ10 (i.e., JAS1, At5g13220) affects plant growth in response to either MeJA treatment or wounding^16^. Furthermore, Shyu *et al.*^43^ have shown that 3*5S:AtJAZ8* plants have greatly increased capacity to repress JA responses. This suggests that the LPIARR motif is an important signal of degradation, but is lacking in *AtJAZ8*, which leads to greater protein stability. Here, those unique features were also manifested by *SmJAZ8*. When over-expressed, its transcript repressed the ability of MeJA to inhibit root growth and blocked the activation of JA-inducible genes. This is probably because, as with *AtJAZ8*, its structure does not contain the LPIARR motif.

Our results also showed that modulating the levels of *SmJAZ8* transcript partially altered the MeJA-responsive accumulation of phenolic acids, which indicated that the JA-induced upregulation of RA and Sal B is mediated by this JAZ repressor.

Stress-induced expression of *SmJAZ8* may prevent excessive activation of JA responses in plants, similar to how *AtJAZ8* and the alternative splice variant of *AtJAZ10* participate in negative feedback control of jasmonate signaling^44^. Therefore, we propose that *SmJAZ8* acts as a repressor that can strongly affect the downstream response to this signal.

### The involvement of *SmJAZ8* in biosynthesis of phenolic acids

One of our goals was to understand the crosstalk between the JA signal pathway and production of phenolic acids. Specifically, we wondered whether blocking transduction, either entirely or partially, of JA signal could alter the accumulation of phenolic metabolites in *S. miltiorrhiza*. In tobacco, only the expression of dominant-negative truncated *JAZ* constructs can interrupt the COI1-JAZ pathway and depress basal levels of nicotine^18^. In *JAZ1∆3A*-transformed *Arabidopsis*, which constitutively expresses truncated AtJAZ1 that lacks the C-terminal Jas-containing region, much less anthocyanin is accumulated following MeJA treatment, while no remarkable differences in levels are measured under normal growing conditions^19^. A common feature underlying JA-regulated biosynthesis of secondary metabolites is the involvement of JAZ proteins along with specific TFs and other components. Further research is needed to elucidate the distinct pathways by which diverse metabolites are activated in different plant species^45^. Here, biosynthesis of total phenolic acids and flavonoids was enhanced in transgenic plants, and antioxidant capacity was also increased. Consistent with a role for JAZ in forming homo- and heterodimers^46^, we found that expression was decreased for some *JAZ* genes, including *SmJAZ1*, *SmJAZ2*, and *SmJAZ3*, which indicated that other pathways exist. As we had predicted, basal expression of *PAL*, *HPPR*, *TAT*, and *RAS* was up-regulated while Sal B accumulated to higher levels in OEB-transgenic plants.

All of our results suggest that crosstalk is possible among individual JAZ proteins, as illustrated by the positive regulatory effect of the SmJAZ8 repressor on phenolic acid accumulations. Furthermore, the high production of secondary metabolites may led to attenuate the JA responses by stably expressed *SmJAZ8*, and induction of its expression in wild-type plants was strongly activated by MeJA from much lower basal levels. Therefore, we propose that these associated metabolite accumulations occur downstream of *SmJAZ8* activation. While the extent of the contribution by this gene to that phenomenon is unclear, it may be part of other pathways. Similar crosstalk within JAZ-regulated secondary metabolism has been described for *Nicotiana attenuata*, where the silencing of NaJAZh significantly reduces nicotine levels^47^. Furthermore, overexpression of *GsJAZ2* in *Glycine soja* leads to enhance plant tolerance with positive regulation of expression of stress-inducible marker genes^48^. Thus, it should also be possible to identify the differentially expressed TFs that are directly integrated with SmJAZ8 or other JAZ proteins to regulate secondary metabolism in *S. miltiorrhiza.* For example, R2R3-MYB factors are involved in modulating many phenolic pathways^49^, such as *AtPAP1*, which induces the accumulation of anthocyanin in *Arabidopsis*^19^ and Sal B in *S. miltiorrhiza*^40^. Because of this, it is tempting to speculate on the existence of a PAP1 in *S. miltiorrhiza* that is controlled by JAZ protein and which could control phenolic metabolism. Future investigations of any relationship between PAP1 and JAZ genes in *S. miltiorrhiza* should improve our understanding about the novel crosstalk in JAZ-regulated secondary metabolism within this model medicinal plant.

## Materials and Methods

### Plant materials and treatments

Seeds of *Salvia miltiorrhiza* Bunge, collected from Shangluo, Shaanxi Province, China, were germinated in vermiculite. When their fourth true leaves appeared, the plantlets were transferred to pots filled with soil (two per pot), then grown in our laboratory’s greenhouse. Temperatures ranged from 24 to 26 °C under a natural. Plants were irrigated with distilled water at 3-d intervals. Prior to the treatment period, all plants were acclimated for one week in a growth chamber under a 16-h photoperiod provided from cool-white fluorescent lamps (25 μmol m^−2^ s^−1^). The six-month-old plants were then randomly assigned to treatment groups. To examine the effect of MeJA (Sigma Chemical Co.), we sprayed the plants with either a 500 μM MeJA solution (100 mL per plantlet) that was mixed with 2% ethanol (v/v) in water (treatment solution) or else a mock (MOCK control) solution that contained only the ethanol/water mixture. After the plants were hermetically sealed in plastic bags for 1 h, they were exposed to open air for 2 h so that any residual ethanol water and MeJA could evaporate from the plants. Samples for transcript profiling were harvested from three biological replicates at 6 h after treatment, then immediately frozen in liquid nitrogen and stored at −80 °C. For metabolite profiling, plantlets from these two treatment groups were harvested at 0, 3, 6, and 12 d, then dried at 30 °C before being pulverized to a fine powder (2-mm mesh) and stored at 4 °C.

To ensure stable asexual reproduction, we surface-sterilized *S. miltiorrhiza* seeds with a 1% (v/v) mercuric chloride solution for 8 min, and with 75% (v/v) ethanol for 25 s. They were then thoroughly washed in sterile water and placed for 7 d at 25 °C in the dark on an MS basal medium containing 0.65% agar. Uniformly germinated seeds were selected and cultured on a fresh MS basal medium containing 0.65% agar and 3% sucrose, as described by Yan and Wang^50^. The MeJA treatment described above was repeated with these sterile plantlets to verify those results. After approximately one month of growth, two leaf blades at the top of each selected stem were removed and placed on another solid MS-based medium via the clipping inoculation technique. These seedlings were then grown for another 3 d before being transferred to a medium supplemented with either MeJA (100 μM final concentration) or the mock solution (ethanol/water mixture). For quantitative real-time PCR (qRT-PCR) analysis, seedlings were harvested at Hour 6 and frozen at −80 °C. Contents of secondary metabolites were monitored over time by HPLC. Root lengths were measured on Day 14, followed by washing with distilled water and scanning at a resolution of 600 dpi. The root phenotypes were examined with WinRHIZO software (Aozuo, China).

Hormone treatments were conducted by spraying the leaves of three-month-old seedlings with 5 mM MeJA, 150 μM gibberellin (GA_3_), or 150 μM paclobutrazol (PAC). Control seedlings received only water. Tissues were sampled after 0, 0.5, 1, 2, 4, 6, 8, 12, and 24 h.

To investigate the effect of mechanical wounding, we crushed the leaf midribs three times with a hemostat, using approximately four leaves per plant to damage approximately 40% of the total area^33^. Samples were collected for analysis after 0, 0.5, 1, 2, 4, and 6 h.

All experiments were repeated at least three times; results from one representative experiment are presented here.

### Transcript profiling

To identify the genes associated with the JA signaling pathway in *S*. *miltiorrhiza*, we used Solexa sequencing to perform digital gene expression (DGE) profile analysis of our treated plants at Day 180. Total RNA was isolated from each sample with RNeasy Plant Mini kits (OMEGA, China) according to the manufacturer’s instructions. The quality and concentration of total RNA were measured by 1.0% agarose gel electrophoresis and spectrophotometric analysis (SHIMADZU UV-2450). The RNA samples >300 ng μL^−1^ with high purity (OD_260/280_ > 1.8, OD_260/230_ > 1.5) underwent transcriptome analysis and qRT-PCR. Digital expression and sequencing were performed at BGI in Shenzhen, China (http://www.genomics.org.cn/). Sequences were compared by conducting a Blastn search with two databases: NCBI and our transcriptome for *S. miltiorrhiza*^24^. All clean tags obtained by filtering out any adaptor-only tags and low-quality tags were annotated based on *S. miltiorrhiza* reference genes (Table S4). For statistical analysis, we used a method previously described by Zhang *et al.*^26^ to identify differentially expressed genes (DEGs) based on false discovery rates (≤0.001), *p-*values of < 0.005, and estimated absolute log^2^ fold-changes of ≥1 in sequence counts across libraries (Figures S4, S5). Significantly enriched metabolic pathways or signal transduction pathways were functionally classified into several categories with KEGG (http://www.genome.jp/kegg/) by comparing those DEGs with the control genome background (Table S5). Pathways with Q values of < 0.05 were considered to be significantly enriched in DEGs.

### Metabolite profiling by GC/MS and LC/MS

All samples were harvested and extracted as described previously by Zhang *et al.*^26^ Air-dried roots from treated and untreated samples (60 mg each) were extracted, derivatized, and prepared for GC/MS using a scaled-down version of a two-phase methanol/chloroform method^51^. For profiling, 1 μL of supernatant was applied to an Agilent 6890N GC-FID and Shimadzu QP 2010 GC/MS (Kyoto, Japan), using a 20:1 split injection ratio. Separations were made on a fused silica capillary Agilent Technology HP-5ms (5% phenyl methyl siloxane) column (30 m × 0.25 mm i.d., 0.25 μm film thickness). The injector and detector temperatures were 250 °C and 200 °C, respectively. The oven temperature was 136 °C for the first 5 min, but was then ramped to 300 °C in 4 °C increments, with a Nitrogen gas linear velocity of 1.56 mL min^−1^ and a data acquisition rate of 20 Hz. Full-scan mass spectra were acquired from 40 to 600 *m*/*z*. Mass spectrometry was performed at source and interface temperatures of 200 °C and 250 °C, respectively, with a voltage of 0.9 kV. Metabolites were identified according to the protocol of Ma *et al.*^52^, which had been adapted by Zhang *et al.*^26^ for use with *S. miltiorrhiza*. We subsequently obtained a peak table of whole samples, including retention times and MS fragment ions (Table S2), by comparing their mass spectra with those from the National Institute of Standards and Technology(NIST, USA) library as well as with standard compounds. No peak was available from the table if its area was zero in more than 80% of the samples. The relative areas of the remaining 16 peaks were mean-centered (Ctr) or pareto-scaled (Par), then evaluated by Principal Component Analysis (PCA) and partial least squares-discriminant analysis (PLS-DA), via the SIMCA-P software program (Umetrics, Umea, Sweden). Independent one-way ANOVAs were used with SPSS (version 13.0) to select variables with VIP (variable importance in the projection, more than 1) *p*-values of < 0.05. These were chosen because we believed any significant differences in compounds between our control and treated groups were evidence of important roles in functional classifications.

For LC/MS, air-dried samples were homogenized in 500 μL of extraction solvent (3:1 methanol:H_2_O) per 100.0 mg of dry weight. The extracting procedure was performed as described by Zhang *et al.*^40^. Afterward, 20 μL of supernatant was applied to an Agilent 1100 HPLC system (Agilent Technologies, Palo Alto, CA, USA) that comprised a quaternary solvent delivery system, on-line degasser, auto-sampler, column temperature controller, and photodiode array detector coupled with an analytical workstation. HPLC was performed on a Phenomenex C_18_ column (5 μm, 250 mm × 4.6 mm) at a flow rate of 1.0 mL min^−1^ (temperature 30 °C). The elution gradient included Solvent A (0.4% acetic acid in water), Solvent B (acetonitrile), and Solvent C (methanol), and followed a profile of 0 min, 95% A, 5% B, 0% C; 5 min, 90% A, 10% B; 25 min, 67% A, 30% B, 3% C; 40 min, 60% A, 35% B, 5% C; 55 min, 49% A, 45% B, 6% C; 60 min, 47% A, 45% B, 8% C; 80 min, 35% A, 55% B, 10% C; 90 min, 25% A, 60% B, 15% C; 95 min, 10% A, 70% B, 20% C; and 100 min, 80% B, 20% C). Linear gradients were used between time points. A PDA was utilized for detecting UV-visible absorption between 250 and 650 nm, with samples measured at 280 nm. Mass spectra were acquired by a Bruker Esquire 6000 ion trap instrument with an ESI source (Bruker Daltonics, Inc., USA). For phenolic acids, the ion chromatograms of negative electrospray ionization were recorded from 0 to 60 min by a mass spectrum detector. The spectrum was scanned and stored from m/z of 100 to 1000. For the tanshinones, the ionization mode was positive (60 ~ 100 min), and spectra were recorded between 100 and 800 *m*/*z*. The drying gas flow was 12 L min^−1^ and the nebulizer pressure was set to 35 psi at a capillary temperature and voltage of 320 °C and 30 V, respectively. The collision-induced dissociation (CID) energy was adjusted to 40 ~ 50%. Metabolites were identified based on their retention times, UV spectra, and mass fragmentation by applying tandem MS analysis and comparing results with known compounds in our laboratory data^26^. As authentic standards, caffeic acid, rosmarinic acid, salvianolic acid B, cryptotanshinone, tanshinone I, and tanshinone IIA were purchased from the National Institute for the Control of Pharmaceutical and Biological Products (Beijing, China).

### Quantitative real-time PCR analysis

To evaluate the reliability of DEGs identified by DGE, we used qRT-PCR to profile candidate genes that might respond to MeJA treatment. Based on the KEGG pathway annotation, 10 genes were selected randomly to compare their expression patterns under different conditions. The cDNA was generated from total RNA using a Revert Aid First Strand cDNA Synthesis Kit (Takara) according to the manufacturer’s instructions. We chose *Actin* as the internal reference gene for normalization because its expression has previously been proven stable in *S. miltiorrhiza*^53^. Relative gene expression was evaluated by using Real Master Mix SYBR Green II dye (Takara, Japan) and the iQ™ 5 Real-time PCR detection system (Bio-Rad, USA), following earlier protocols^40^. The primers used for detection are listed in Table S7.

### Contents of targeted phenolic compounds determined by HPLC

We targeted two phenolic compounds to examine the effects of MeJA on accumulations of secondary metabolites. Seedlings exposed to MeJA treatment for 0, 3, 6, 9, or 12 d were sampled and ground into powders, which were then extracted as described above. The short (40-min) procedure for HPLC analysis was the same as for metabolite profiling.

### Isolation and sequence analysis of *SmJAZs*

To identify members of the *JAZ* gene family within *S. miltiorrhiza*, we screened the DGE and transcriptome databases that had been constructed and conserved by our laboratory^24^. Sequences were then assembled into putative *JAZ* genes by using DNAStar SeqMan 5.0 software. Those assemblies were compared against the nonredundant peptide database at the National Center for Biotechnology Information (NCBI) (http://www.ncbi.nlm.nih.gov). The performance of four fragments was more similar to other JAZ genes from *Arabidopsis thaliana*, and contained two conserved ZIM and JAZ motifs. Primers were designed (Table S7) to clone the corresponding sequences. If necessary, the primers were redesigned until a clear PCR product of estimated length could be amplified from the cDNA template. All PCR products were purified and cloned into a pGEM-T easy vector (Promega, USA) before sequencing. At least three independent clones were fully sequenced for each type of insert.

To test expression in response to hormones, we also designed gene-specific primer pairs (Table S7). Total RNA was also extracted from the roots, stems, leaves, and flowers of two-year-old plants to investigate transcription patterns. That age was used so that we could obtain flower organs. All reactions were performed under the qRT-PCR conditions described above.

The sequence of *SmJAZ8*, a novel JAZ gene, was also amplified by PCR, using total DNA as template. Genomic DNA was isolated from the leaves of three-month-old seedlings per the modified cetyl-trimethyl ammonium bromide method^54^. Combined with the cDNA sequence, a diagram of exon/intron structures was obtained using public online software from the Gene Structure Display Server (http://gsds.cbi.pku.edu.cn), which exhibited both exon positions and conserved domains. The full-length sequence of its translated putative protein was aligned with those of other JAZ proteins by the ClustalW program, and a phylogenetic tree was built using bioinformatics software MEGA 5.0 (Figure S6), the maximum parsimony (MP) method, and a bootstrap test (1,000 replicates).

### Plant transformation

To generate transgenic plants, we amplified the coding regions of *SmJAZ8* from pGEM-T-SmJAZ8 (described above). The sequence was inserted into the pKANNIBAL vector, placed under the CaMV 35S promoter. The construct was restricted by *Not* I, and the digested fragment was ligated to the pART-27 vector. The final recombined plasmid was introduced into *Agrobacterium tumefaciens* EHA105, and transgenic S. *miltiorrhiza* plants were generated by the leaf-disc method.^50^ Transformation was verified by screening with kanamycin and cephalosporin. Our controls were either wild-type plants or those transformed with only the binary vector pKannibal-pART27. Transgenic lines were confirmed by PCR screening using genomic DNA, and qRT-PCR was used to select the best overexpression transgenic lines — OEB-2, OEB-4, OEB-5, and OEB-7. One-month-old plants were used for preliminary quantification of phenolic acids as well as investigations of total flavonoids and the MeJA-responsive phenotype. For HPLC analysis of the phenolic compounds, those transgenics and the control plants were transferred into soil and grown in a greenhouse for at least one month.

### Analysis of total phenolics, total flavonoids, and antioxidant capacity

The roots from one-month-old plants were air-dried at room temperature. The methodology for extracting and determining total phenolics and flavonoids followed procedures described by Zhang *et al.*^40^.

Total antioxidant activities were evaluated for trolox equivalent antioxidant capacity (TEAC), based on our previously published protocols^26^.

For assaying the scavenging of free radicals in methanolic extracts, we utilized the DPPH method of Zhang *et al.*^55^ with slight modifications. Different dilutions of the extracts (2 mL; 0.1–1.5 mg mL^−1^) were added to 1 mL of a 0.004% methanol solution of DPPH. The positive reference was vitamin C (V_C_). After 30 min of incubation at room temperature, scavenging activity was spectrophotometrically measured at 517 nm, and was calculated by the following formula:


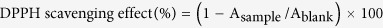


where A_blank_ was the absorbance of the control reaction and A_sample_ was the absorbance of the test sample.

The EC_50_ value was used to represent the antioxidant capacity of the crude extracts and V_C_ standard, i.e., the effective concentration at which DPPH free radicals were depleted by 50%.

## Additional Information

**How to cite this article**: Ge, Q. *et al.* Combination of transcriptomic and metabolomic analyses reveals a JAZ repressor in the jasmonate signaling pathway of *Salvia miltiorrhiza*. *Sci. Rep.* **5**, 14048; doi: 10.1038/srep14048 (2015).

## Supplementary Material

Supplementary Information

## Figures and Tables

**Table 1:** Effects of MeJA on root morphology for Control and *SmJAZ8*-overexpressing plants (n = 15).

**Line**	**Condition**	**Max length (cm)**	**Total length (cm)**	Average diameter(mm)	**Number of tips**	Number of lateralroots
Control	MOCK	7.55 ± 0.46	48.72 ± 4.70	0.60 ± 0.03	29.25 ± 5.40	55.75 ± 11.77
	MeJA	5.22 ± 0.48**	21.67 ± 3.07**	0.78 ± 0.10**	14.75 ± 5.63*	26.25 ± 5.15**
OEB-5	MOCK	6.03 ± 0.98	42.56 ± 1.73	0.65 ± 0.05	34.50 ± 6.58	72.75 ± 18.42
	MeJA	4.60 ± 0.55	25.52 ± 5.37*	0.93 ± 0.09**	20.00 ± 4.20	59.67 ± 8.80
OEB-7	MOCK	6.26 ± 0.83	50.60 ± 6.88	0.62 ± 0.03	28.67 ± 7.41	52.33 ± 9.10
	MeJA	5.57 ± 0.82	32.40 ± 8.38	0.76 ± 0.06	24.67 ± 4.92*	59.67 ± 8.80

*significant at *p* < 0.05; **highly significant at *p* < 0.01.

**Table 2:** Changes in RA and Sal B accumulations by transgenic lines under MeJA treatment.

**Line**	**Condition**	**RA (mg g^−1^DW)**	**Sal B (mg g^−1^DW)**
Control	MOCK	29.33 ± 0.65	11.43 ± 0.37
	MeJA	41.98 ± 0.65**	16.97 ± 0.21**
OEB-5	MOCK	33.29 ± 2.12	15.92 ± 0.95
	MeJA	36.29 ± 2.29	15.82 ± 1.03
OEB-7	MOCK	27.52 ± 2.00	9.67 ± 0.54
	MeJA	31.02 ± 0.99	13.87 ± 0.21*

*significant at *p* < 0.05; **highly significant at *p* < 0.01.
