# Supplementary material for: Combination of transcriptomic and metabolomic analyses reveals a JAZ repressor in the jasmonate signaling pathway of Salvia miltiorrhiza
Source: Sci Rep. 2015 Sep 21;5:14048. doi: 10.1038/srep14048 (PMC4585666; doi:10.1038/srep14048)
Supplement: Supplementary Information [file srep14048-s1.doc]

**Combination of transcriptomic and** **metabolomic analyses reveals a JAZ repressor in the jasmonate signaling pathway of** ***Salvia miltiorrhiza***

Qian Ge^1*^, Yuan Zhang^1*^, Wen-Ping Hua^1^, Yu-Cui Wu^1^, Xin-Xin Jin^1^, Shuang-Hong Song^2^, Zhe-Zhi Wang^1^

^1^ Key Laboratory of the Ministry of Education for Medicinal Resources and Natural Pharmaceutical Chemistry, National Engineering Laboratory for Resource Development of Endangered Crude Drugs in Northwest of China, College of Life Sciences, Shaanxi Normal University, Xi’an, China; ^2^ Co-Innovation Center for Qinba regions’ sustainable development, College of Life Sciences, Shaanxi Normal University, Xi’an, China

*These authors contributed equally to this study.

Correspondence and requests for materials should be addressed to Z.Z.W. (email:zzwang@snnu.edu.cn).


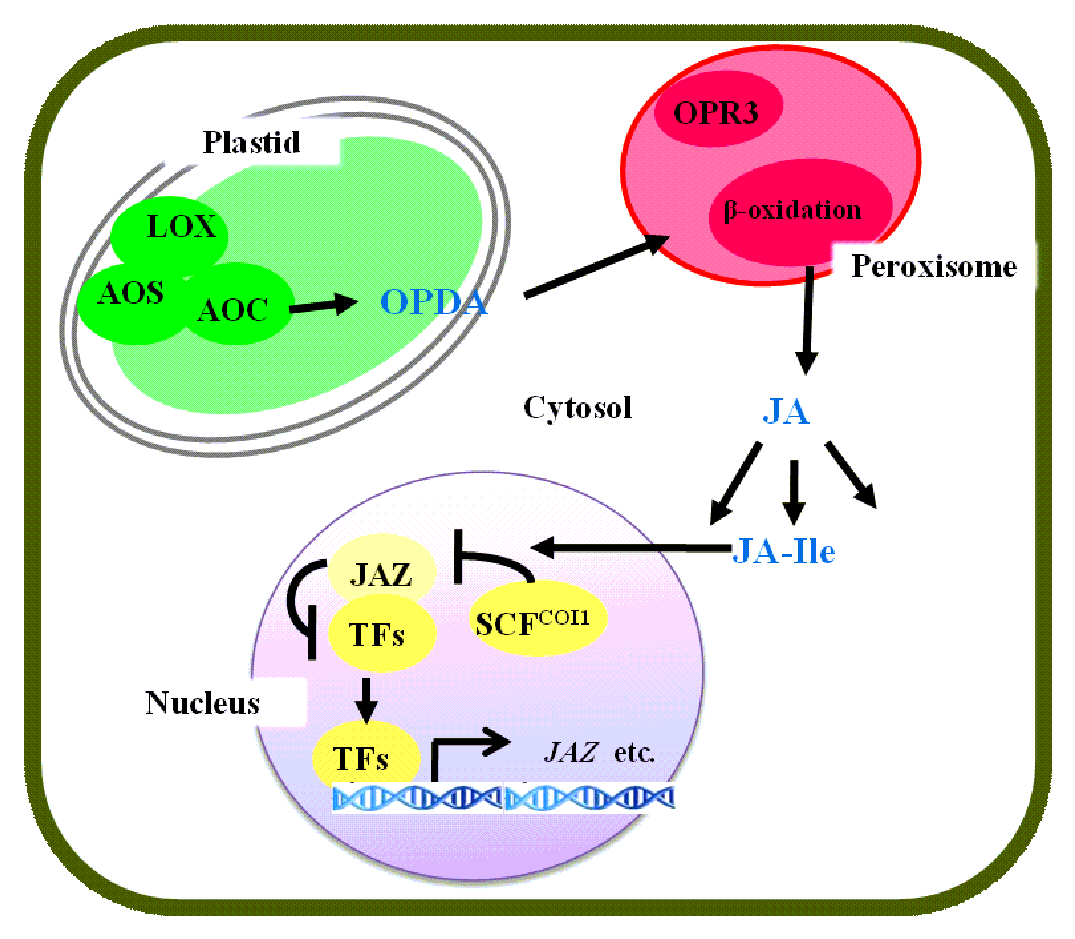


Figure S1. Cellular compartments for JA synthesis and signaling.[**^1^**](#_ENREF_1) AOC, allene oxide cyclase; AOS, alleneoxidesynthase; COI1, coronatine insensitive1; JAR, jasmonyl isoleucine conjugate synthase; JAZ, jasmonate ZIM domain proteins; LOX, lipoxgenase; OPR3, OPDA reductase 3; OPDA, cis-(+)-12-oxophytodienoic acid; TFs, transcription factors.

**
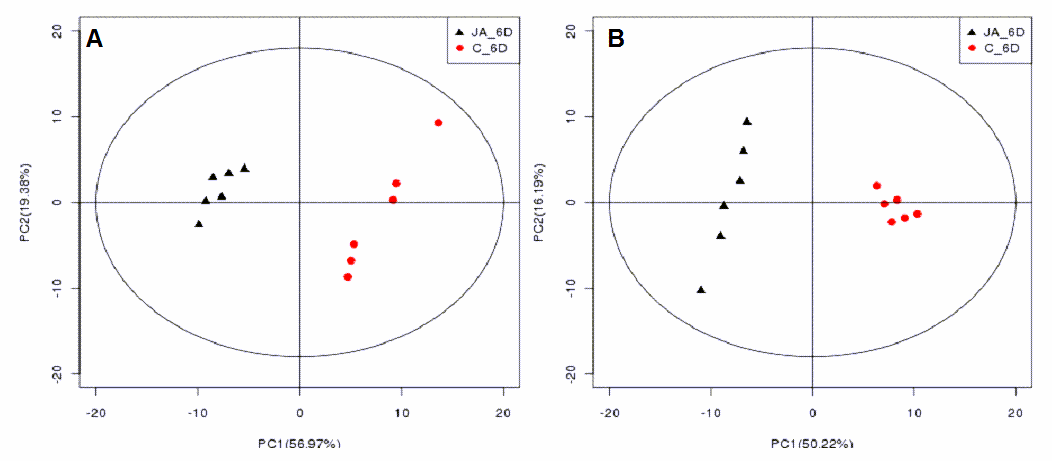
**

Figure S2. Score plots derived via Principal Component Analysis (PCA) from GC/MS (A) and LC/MS (B) data sets of control and MeJA treatment sample. Variation explained in X-matrix (R^2^X) was 0.764 (GC/MS) and 0.996 (LC/MS); predictive ability (Q^2^X) of each data set was 0.598 and 0.999, respectively.


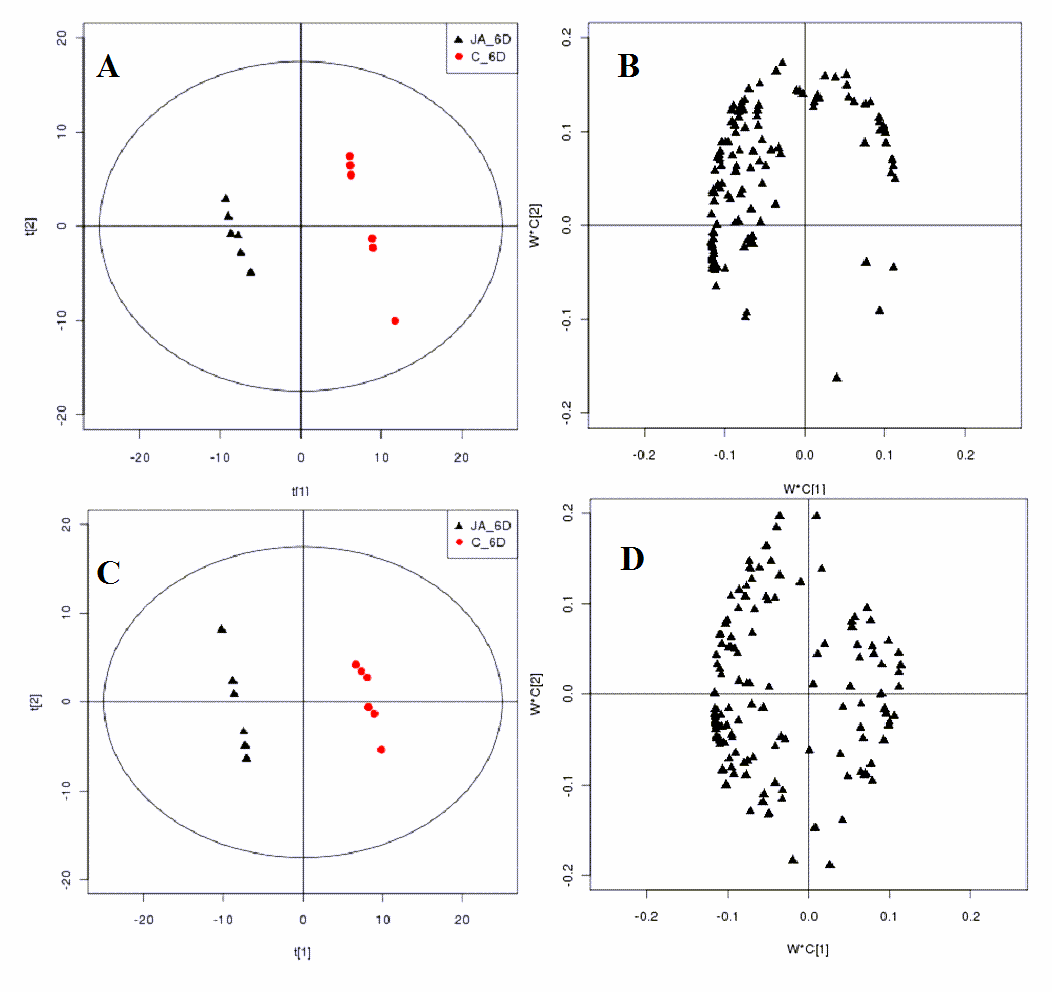


Figure S3. PLS-DA score plots (A, C) and loading plots (B, D) derived from GC/MS (A, B) and LC/MS (C, D) data sets of control and MeJA treatment sample. Variation explained in Y-matrix (R^2^Y) was 0.999 (GC/MS) and 0.999 (LC/MS); predictive ability (Q^2^Y) was 0.996 and 0.991, respectively.


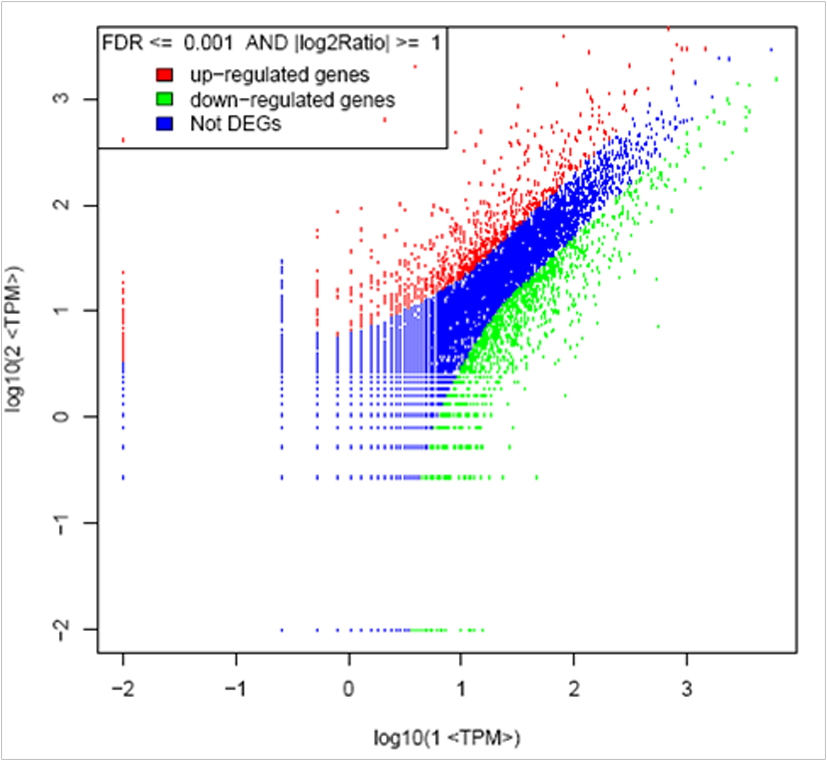


Figure S4. Scatter plot of differentially expressed genes in *S. miltiorrhiza* induced by MeJA and control. TPM, transcripts per million (normalized expression level of genes).


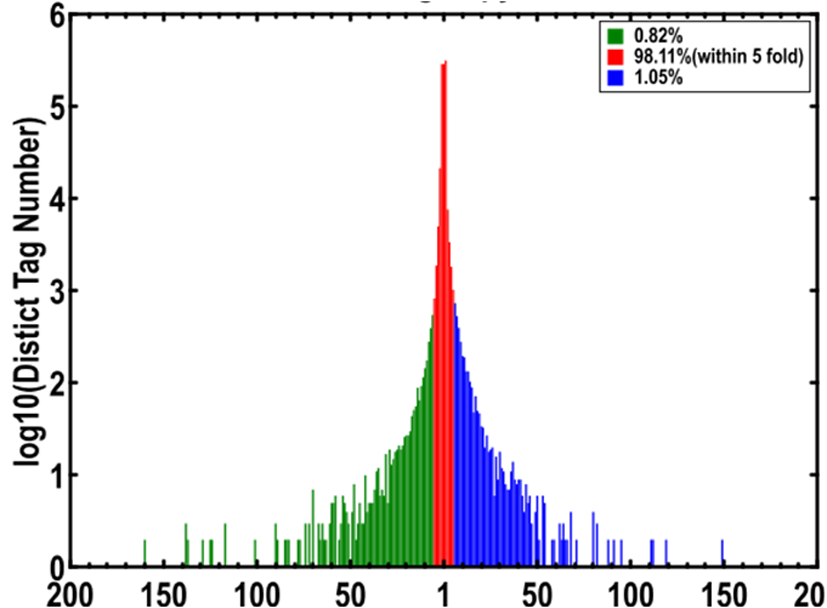


Figure S5. Differentially expressed tags in JA library.

x-axis, fold-change; y-axis, number of unique tags (log10). Differentially accumulating unique tags with a 5-fold difference between libraries are shown in red (98.11%). Blue (1.05%) and green (0.82%) regions represent unique tags that are up- and down-regulated by >5-fold, respectively.


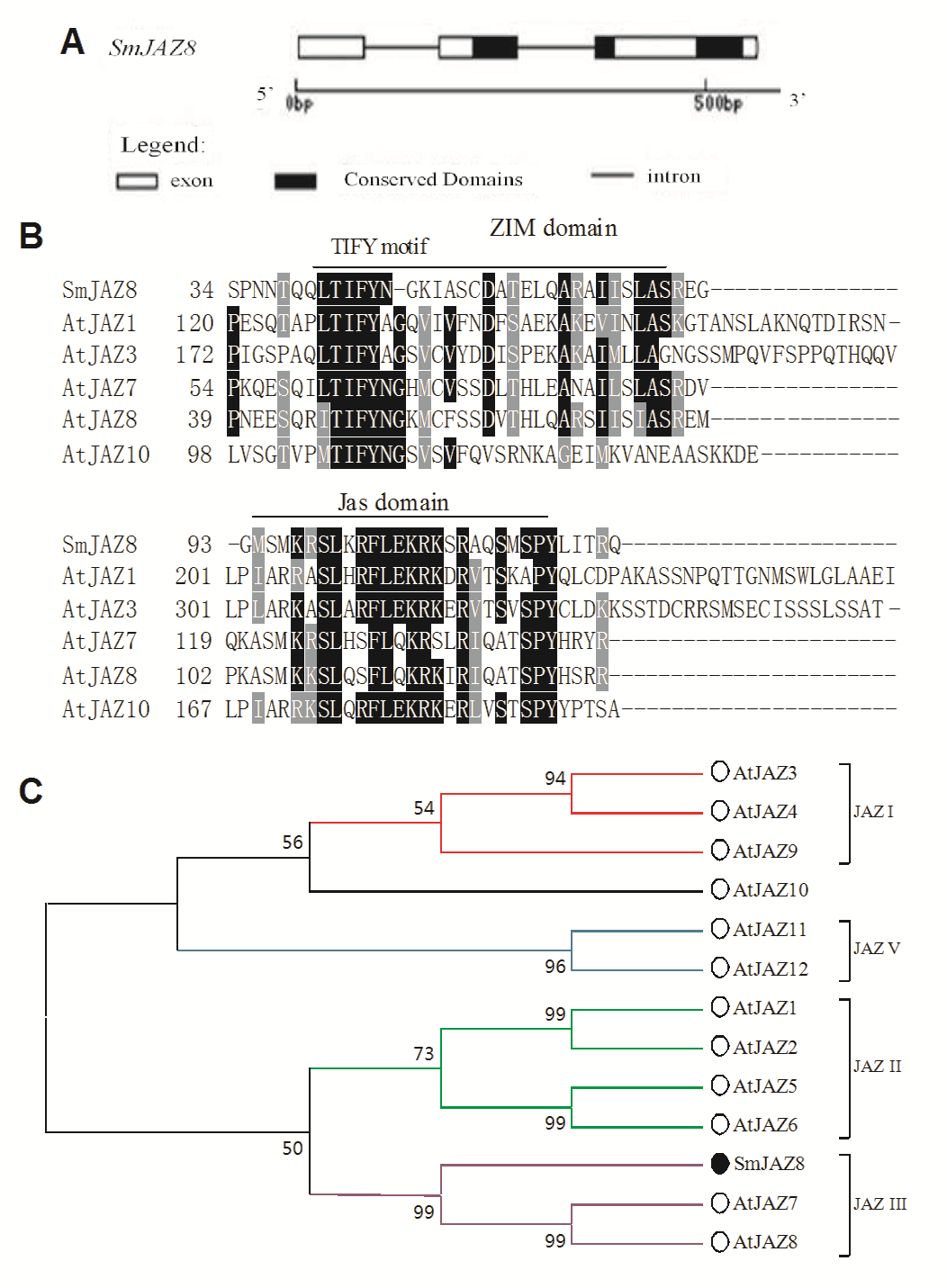


Figure S6. Details of SmJAZ8 structure and phylogenetic analysis. A, Exon/intron structures of *SmJAZ8*. Exons are denoted by white boxes,. Black lines connecting two exons represent introns. Conserved domains are ZIM and Jas motifs, respectively. B, Deduced amino acid sequences of the ZIM and Jas motifs in SmJAZ8 and similar corresponding Arabidopsis homologs (AtJAZ1, AtJAZ3, AtJAZ7, AtJAZ8, and AtJAZ10) are aligned. C, Phylogenetic tree of deduced amino acid sequence for SmJAZ8 protein and JAZ homologs from other plants, constructed according to neighbor-joining method, using MEGA 4 software. Values on each node are percentage of bootstrap values (only values >50 are shown). GenBank Accession Numbers for JAZ homologous proteins in phylogenetic tree and multiple sequence alignment are Q9LMA8.1(AtJAZ1), Q9LVI4.1(AtJAZ3), O64687.1 (AtJAZ7), Q8LBM2.1(AtJAZ8), and Q93ZM9.1 (AtJAZ10).


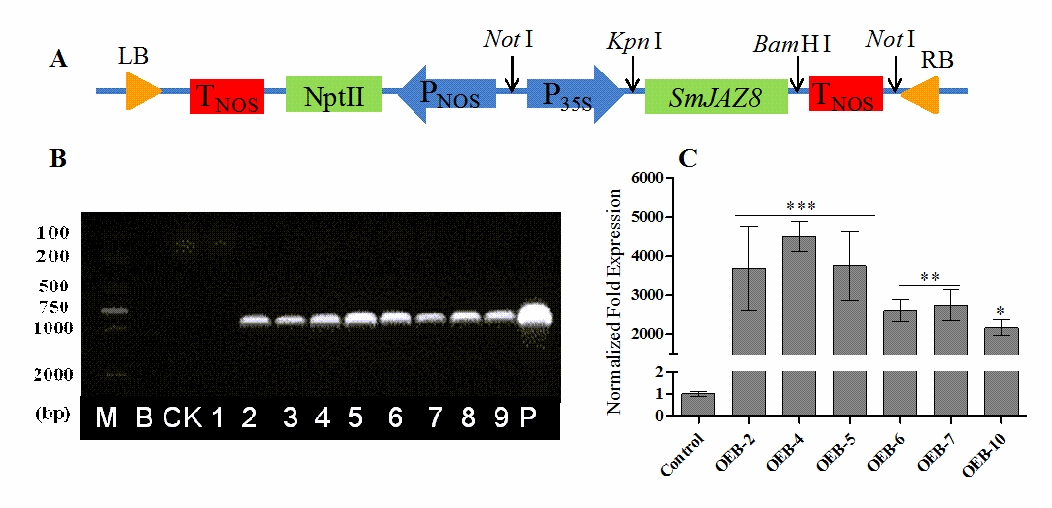


Figure S7. Generation of transgenic *Salvia miltiorrhiza* plants.

A, Schematic representation of a construct used for *Agrobacteriumtumefaciens*-mediated transformation of the *SmJAZ8* gene.

B, PCR results from OEB-transgenic and control lines. M, DL2000 DNA Marker, band sizes from top: 100, 250, 500, 750, 1000, and 2000 bp; B, no template control; Lane 1, control; Lanes 2-13, transgenic lines; Lane 14, positive control (JAZ8 vector).

C, Real-time quantitative PCR analysis of *SmJAZ8* expression. All data are means of 3 replicates, with error bars indicating SD. CK, indicate wild-type, which was untransformed lines; OEB, indicate transgenic lines (**P*<0.05, ** *P*<0.01, *** *P*< 0.001).

Table S1 Effects of MeJA on the phenotype features of *S.miltiorrhiza* roots（n=15）

|  | **Max length （cm）** | **Total length （cm）** | **Average diameter （mm）** | **Tip number** | **Number of lateral roots** |
| --- | --- | --- | --- | --- | --- |
| **MOCK** | 8.34±1.36 | 90.21±25.80 | 0.56±0.03 | 56±16 | 122±47 |
| **MeJA** | 4.09±1.64^**^ | 14.36±8.27^**^ | 0.72±0.09^**^ | 7±3^**^ | 9±5^**^ |

** indicates extremely significantly (*P*<0.01)

Table S2 Differential metabolites between *S. miltiorrhiza* induced by MeJA and control based on GC-MS data

| *Differential metabolites* | *Retention time*  *(min)* | *VIP ^A^* | *MS fragment ions*  *(m/z)* | *Change fold*  *(JA / C) ^B^* |
| --- | --- | --- | --- | --- |
| threonine | 5.26 ± 0.02 | 1.31 | 73, 117, 218, 219, 101 | 3.56 |
| malic acid | 7.71 ± 0.03 | 1.02 | 73, 147, 233, 75, 245, 74 | 2.62 |
| proline | 8.53 ± 0.15 | 1.35 | 156, 73, 147, 157, 45, 75 | 4.55 |
| butanoic acid | 8.72 ± 0.03 | 1.23 | 174, 73, 147, 75, 175, 304 | 0.66 |
| asparagine | 10.96 ± 0.13 | 1.36 | 73, 116, 231, 132, 75, 147, 45 | 8.22 |
| glutamine | 11.20 ± 0.04 | 1.29 | 73, 156, 155, 146, 75, 147 | 1.65 |
| D-fructose | 18.35 ± 0.08 | 1.37 | 73, 103, 217, 307, 147, 74 | 10. 84 |
| D-ribose | 18.62 ± 0.04 | 2.21 | 73, 103, 307, 217, 147, 189 | 8.25 |
| glucose oxime | 18.81 ± 0.12 | 1.93 | 73, 319, 205, 147, 320, 218 | 6.75 |
| D-mannitol | 18.92 ± 0.55 | 1.37 | 73, 319, 205, 147, 103, 217 | 10.13 |
| galactose oxime | 19.38 ± 0.16 | 2.36 | 73, 319, 205, 147, 103 | 6.56 |
| palmitic acid | 21.61 ± 0.06 | 2.08 | 73, 75, 117, 43, 132, 313 | 1.51 |
| *α*-D-glucopyranose,  *β*-D-fructofuranosyl  (sucrose) | 34.92 ± 0.02 | 2.35 | 361, 73, 362, 217, 147, 363 | 2.14 |
| stearic acid | 36.65 ± 0.12 | 2.01 | 73, 117, 75, 132, 43, 341 | 4.26 |
| 4-O-α-D-glucopyranosyl  -D-glucose  (maltose) | 39.01 ± 0.03 | 2.35 | 217, 73, 204, 361, 147, 129 | 26.56 |
| *β*-D-glucopyranose,  *β*-D-galactopyranosyl  (lactose) | 40.90 ± 0.08 | 1.29 | 204, 73, 191, 217, 205 | 1.21 |

^A^ Variable Importance (VIP) was obtained from PLS-DA with a threshold of 1.0;

^B^ JA/C > 1 indicates a relatively higher concentration present in *S. miltiorrhiza* induced by MeJA, while JA/C < 1 means a relatively lower concentration as compared to the controls;

JA: *S. miltiorrhiza* induced by MeJA, C: control;

*: metabolites identified only by NIST library (similarity > 90%).

Table S3 Differential metabolites between the *S. miltiorrhiza* induced by MeJA and control based on LC-MS data

| *Differential metabolites* | *Retention time*  *(min)* | *VIP ^A^* | *[M-H]^-^*  *(m/z)* | *MS^(n)^ fragment ions*  *(m/z)* | *Change fold*  *(JA / C) ^B^* |
| --- | --- | --- | --- | --- | --- |
| protocatechuic acid | 11.18 ± 0.04 | 1.08 | 153 | 109 | 0.59 |
| protocatechuic aldehyde | 13.51 ± 0.06 | 1.38 | 137 | 109 | 3.60 |
| caffeic acid | 16.79 ± 0.02 | 1.42 | 179 | 135 | 1.26 |
| ferulic acid | 18.36 ± 0.08 | 1.48 | 193 | 150, 179 | 4.67 |
| salvianolic acid F | 19.54 ± 0.08 | 1.42 | 313 | 269, 159 | 3.84 |
| salvianolic acid D | 20.57 ± 0.09 | 1.48 | 471 | 175, 373, 197 | 1.56 |
| salvianolic acid G | 21.95 ± 0.03 | 1.66 | 339 | 295, 321 | 3.25 |
| salvianolic acid E | 23.72 ± 0.08 | 1. 96 | 717 | 519, 321, 537 | 4.62 |
| rosmarinic acid | 25.02 ± 0.11 | 2.29 | 359 | 161, 197, 179 | 1.36 |
| lithospermic acid | 26.58 ± 0.12 | 1. 62 | 537 | 493, 295 | 1.64 |
| salvianolic acid B | 28.72 ± 0.18 | 3.27 | 717 | 519, 339 | 1.89 |
| iaosalvianolic acid B | 31.77 ± 0.28 | 1.54 | 717 | 519 | 0.36 |
| salvianolic acid L | 32.23 ± 0.08 | 1.39 | 717 | 519, 501 | 2.46 |
| salvianolic acid A | 32.93 ± 0.05 | 1.92 | 493 | 295, 313 | 18.26 |
| tanshinone II-B | 68.92± 0.04 | 1.35 | 311 | 293, 283, 275 | 1.96 |
| tanshinone I | 77.84± 0.22 | 1.96 | 277 | 249, 231 | 2.05 |
| cryptotanshinone | 78.49± 0.08 | 2.05 | 297 | 279, 251 | 1.38 |
| dihydrotanshinone IIA | 84.13± 0.16 | 1.66 | 279 | 261, 233 | 1.69 |
| tanshinone IIA | 90.92± 0.06 | 1.79 | 295 | 277, 249 | 1.33 |

^A^ Variable Importance (VIP) was obtained from PLS-DA with a threshold of 1.0;

^B^JA/C> 1 indicates a relatively higher concentration present in *S. miltiorrhiza*induced by MeJA, while JA/C< 1 means a relatively lower concentration as compared to thecontrols;

JA: *S. miltiorrhiza*induced by MeJA, C: control;

*: metabolites identified only by MS^(n)^ fragment ions.

Table S4 Categorization and abundance of tags

| *Summary* |  | *Control* | *MeJA* |
| --- | --- | --- | --- |
| Raw tag | Total | 3843001 | 3787000 |
|  | Distinct tag | 272643 | 203838 |
| Clean tag | Total number | 3819543 | 3763892 |
|  | Distinct tag number | 261601 | 194228 |
| All tag mapping to gene | Total number | 1810024 | 1826424 |
|  | Total % of clean tag | 47.39% | 48.52% |
|  | Distinct Tag number | 79169 | 71830 |
|  | Distinct Tag % of clean tag | 30.26% | 36.98% |
| Unique tag mapping to gene | Total number | 1782177 | 1803454 |
|  | Total % of clean tag | 46.66% | 47.91% |
|  | Distinct Tag number | 78193 | 71021 |
|  | Distinct Tag % of clean tag | 29.89% | 36.57% |
| All tag-mapped genes | number | 25251 | 23298 |
|  | % of ref genes | 27.97% | 25.80% |
| Unambiguous Tag-mapped Genes | number | 24702 | 22859 |
|  | % of ref genes | 27.36% | 25.32% |
| Unknown tag | Total number | 43070 | 34047 |
|  | Total % of clean tag | 1.13% | 0.90% |
|  | Distinct Tag number | 4887 | 2470 |
|  | Distinct Tag % of clean tag | 1.87% | 1.27% |

Clean tags: tags after filtering dirty tags (low quality tags) from raw data;

Distinct tags: different kinds of tags;

Unique tags: the reminder clean tags after removing tags mapped to reference sequences from multiple genes.

**Table S5** List of enriched pathways based on the KEGG database

| *Pathway term* | *Pathway ID* | *DEGs tested* | *Q value* |
| --- | --- | --- | --- |
| Metabolic pathways | ko01100 | 288 | 8.65311E-07 |
| Biosynthesis of phenylpropanoids | ko01061 | 71 | 0.0001 |
| Biosynthesis of plant hormones | ko01070 | 79 | 0.0005 |
| Glycine, serine and threonine metabolism | ko00260 | 19 | 0.0014 |
| Nitrogen metabolism | ko00910 | 19 | 0.0016 |
| Flavonoid biosynthesis | ko00941 | 24 | 0.0016 |
| Phenylalanine, tyrosine and tryptophan biosynthesis | ko00400 | 15 | 0.0017 |
| Biosynthesis of alkaloids derived from shikimate pathway | ko01063 | 46 | 0.0018 |
| Phenylpropanoid biosynthesis | ko00940 | 42 | 0.0018 |
| alpha-Linolenic acid metabolism | ko00592 | 16 | 0.0111 |
| Linoleic acid metabolism | ko00591 | 8 | 0.0163 |
| Metabolism of xenobiotics by cytochrome P450 | ko00980 | 12 | 0.0202 |
| Ascorbate and aldarate metabolism | ko00053 | 15 | 0.0266 |
| Photosynthesis - antenna proteins | ko00196 | 7 | 0.0266 |
| Butanoate metabolism | ko00650 | 13 | 0.0279 |
| Tyrosine metabolism | ko00350 | 14 | 0.0333 |
| Arginine and proline metabolism | ko00330 | 17 | 0.0333 |
| beta-Alanine metabolism | ko00410 | 11 | 0.0337 |
| Lysine degradation | ko00310 | 12 | 0.0337 |
| Alanine, aspartate and glutamate metabolism | ko00250 | 15 | 0.0337 |
| Peroxisome | ko04146 | 18 | 0.0351 |
| Fatty acid metabolism | ko00071 | 14 | 0.0361 |
| Benzoxazinoid biosynthesis | ko00402 | 8 | 0.0404 |
| Isoquinoline alkaloid biosynthesis | ko00950 | 8 | 0.0404 |

Pathways with Q value < 0.05 are significantly enriched.

**Table S6** List of active ingredient and JA biosynthesis related DEGs changed between *S. miltiorrhiza* induced by MeJA and control

| Gene name | Unigene ID | Putative function | JA/C value |
| --- | --- | --- | --- |
| ***Core phenylpropanoid pathway*** | | | |
| *PAL1* | Unigene41245 | phenylalanine ammonialyase | 3.35 |
| *PAL2* | Unigene52119 | phenylalanine ammonialyase | 3.63 |
| *C4H* | Unigene53935 | cinnamate 4-hydroxylase | 2.72 |
| *4CL1* | Unigene54261 | 4-coumarateCoA ligase | 4.00 |
| *4CL2* | Unigene16323 | 4-coumarateCoA ligase | 3.14 |
| *RAS* | Unigene55331 | rosmarinic acid synthase | 3.13 |
| *CYP98A14* | Unigene56687 | cytochrome P450 enzymes | 9.56 |
| *CPR* | Unigene55890 | cytochrome P450 reductase | 27.00 |
| ***Lignin pathway*** | | | |
| *HCT* | Unigene30262 | hydroxycinnamoylCoA:shikimatehydroxycinnamoyltransferase | 6.92 |
| *C3'H* | Unigene56687 | *p*-coumaroylshikimate 3'-hydroxylase | 9.56 |
| *COMT1* | Unigene15683 | caffeic acid *O*-methyltransferase | 0.52 |
| *COMT2* | Unigene35594 | caffeic acid *O*-methyltransferase | 0.25 |
| *CCoAMT* | Unigene39895 | caffeoylCoA *O*-methyltransferase | 0.45 |
| *F5H* | Unigene16558 | ferulate 5-hydroxylase | 3.72 |
| *CCR1* | Unigene53802 | cinnamoylCoA reductase | 0.65 |
| *CCR2* | Unigene21918 | cinnamoyl CoA reductase | 2.42 |
| *CAD* | Unigene37246 | cinnamyl alcohol dehydrogenase | 3.04 |
| *GT1* | Unigene3653 | UDP-glucose:anthocysnin 5-*O*-glucosyltransferase | 0.47 |
| *GT2* | Unigene52094 | UDP-glucose:glucosyltransferase | 0.45 |
| ***Tyrosine pathway*** | | | |
| *TAT1* | Unigene56449 | tyrosine aminotransferase | 3.10 |
| *TAT2* | Unigene38332 | tyrosine aminotransferase | 4.03 |
| *HPPR* | Unigene6607 | hydroxyphenylpyruvatereductase | 5.57 |
| ***Flavonoid pathway*** | | | |
| *CHI* | Unigene5 | chalcone isomerase | 8.72 |
| *F3H* | Unigene54426 | flavanone 3-hydroxylase | 1.27 |
| *FLS* | Unigene33659 | flavonol synthase | 35.69 |
| *F3'H* | Unigene2612 | flavanone 3’-hydroxylase | 0.13 |
|  | Unigene56687 | flavanone 3’-hydroxylase | 9.56 |
| *DFR* | Unigene51780 | dihydroflavonol reductase | 4.22 |
| *LAR* | Unigene53731 | leucocyanidin reductase | 0.10 |
| *CHS* | Unigene36651 | 3-ketoacyl-CoA synthase | 0.11 |
| ***JA biosynthesis pathway*** | | | |
| *LOX* | Unigene56748 | lipoxygenase | 1.04 |
|  | Unigene56388 | lipoxygenase | 2.76 |
|  | Unigene56655 | lipoxygenase | 133.00 |
|  | Unigene56096 | lipoxygenase | 10.04 |
|  | Unigene29305 | lipoxygenase | 5.25 |
| *AOS* | Unigene53543 | allene oxide synhase | 2.04 |
| *AOC* | Unigene55396 | allene oxide cyclase | 26.98 |
| *AOC1* | Unigene55800 | allene oxide cyclase | 5.52 |
| *OPR3* | Unigene55172 | 12-oxophytodienoate reductase 3 | 3.96 |
| *ACX* | Unigene56715 | acyl-CoA-oxidase | 1.54 |
| *JAR* | Unigene56764 | jasmonoyl isoleucine conjugate synthase1 | 0.30 |
| ***JA signalling pathway*** | | | |
| *COI1* | Unigene37104 | coronatine insensitive1 | 0.13 |
| *JAZ* | Unigene41599 | jazmonate Zim domain proteins | 2.41 |
|  | Unigene55665 | jazmonate Zim domain proteins | 98.12 |
|  | Unigene53185 | jazmonate Zim domain proteins | 15.68 |
|  | Unigene9545 | jazmonate Zim domain proteins | 96.06 |
|  | **Unigene36977** | **jazmonate Zim domain proteins** | **4.62** |
|  | Unigene24579 | jazmonate Zim domain proteins | 0.89 |
| *TOPLESS* | Unigene33004 | TOPLESS protein(TPL) | 0.45 |
|  | Unigene33546 | TPL-related proteins | 0.02 |
|  | Unigene37662 | TPL-related proteins | 0.84 |
| *NINJA* | Unigene37660 | novel interactor of JAZ | 0.02 |
| *MYC* | Unigene37277 | basic helix-loop-helix transcription factor | 27.00 |
|  | Unigene54297 | basic helix-loop-helix transcription factor | 1.54 |
|  | Unigene27154 | basic helix-loop-helix transcription factor | 8.19 |
| *MYB* | Unigene48545 | transcription factor PAP1 | 14.72 |

JA/C > 1 indicates the gene was upgruduared in *S. miltiorrhiza* induced by MeJA, while JA/C < 1 means the gene was downgruduared as compared to the controls; JA: *S. miltiorrhiza* induced by MeJA, C: control.

Table S7 Primers used in experimental procedures.

| *Primer* | *Sequence* |
| --- | --- |
| **Primers used for real-time quantitative PCR** | |
| SmPAL2-Q-F | GGCGGCGATTGAGAGCAGGA |
| SmPAL2-Q-R | ATCAGCAGATAGGAAGAGGAGCACC |
| SmC4H-Q-F | CCAGGAGTCCAAATAACAGAGCCG |
| SmC4H-Q-R | GCCACCAAGCGTTCACCAAGAT |
| Sm4CL1-Q-F | TCGCCAAATACGACCTTTCC |
| Sm4CL1-Q-R | TGCTTCAGTCATCCCATACCC |
| SmRAS-Q-F | CCAAAGTCAATTATGCCAAGGG |
| SmRAS-Q-R | GTCGGATAGGTGGTGCTCGT |
| SmCCoAMT-Q-F | GGCATCAGGAGGTTGGGC |
| SmCCoAMT-Q-R | GGCATCAGGAGGTTGGGC |
| SmCCR1-Q-F | TGCAAGGGGAAGGCACGTAT |
| SmCCR1-Q-R | GGATGGGGCCAAGAAGCTGA |
| SmCAD-Q-F | CCCCAAAATCATCCCCACTT |
| SmCAD-Q-R | TCATGGGGCTGTAGGTGGTG |
| SmTAT1-Q-F | CAACTGCTGGTCTTCCACAAAC |
| SmTAT1-Q-R | GCGAGCCAAAACGGACA |
| SmF3H-Q-F | TCAGGCTTTGAGCAATGGGAAGT |
| SmF3H-Q-R | TGAGTCGCATGGGCAGAGGAA |
| SmJAZ-Q-F* | TGTGATGCTACAGAACTTCAGGCT |
| SmJAZ-Q-R | GCGACATTGATTGAGCCCTACT |
| SmACTIN-Q-F | AGGAACCACCGATCCAGACA |
| SmACTIN-Q-R | GGTGCCCTGAGGTCCTGTT |
| SmJAZ1-Q-F | GAGTGAGCCGAAACCAGAAAA |
| SmJAZ1-Q-R | GGAAGGATAGCTGGTGGCTAAA |
| SmJAZ2-Q-F | CTCAGGCAGAGTGTTGGTGTT |
| SmJAZ2-Q-R | TAGAGGCGTTGGATGAAATGG |
| SmJAZ3-Q-F | ACGGTGACAACATACGGAGACA |
| SmJAZ3-Q-R | TCGCCTTAGGAGCCACTGAAT |
| SmJAZ8-Q-F | TGTGATGCTACAGAACTTCAGGCT |
| SmJAZ8-Q-R | GCGACATTGATTGAGCCCTACT |
| SmPAL1-Q-F | GATAGCGGAGTGCAGGTCGTAC |
| SmPAL1-Q-R | CGAACTAGCAGATTGGCAGAGG |
| SmHPPR-Q-F | TGACTCCAGAAACAACCCACATT |
| SmHPPR-Q-R | CCCAGACGACCCTCCACAAG |
| SmHCT-Q-F | GGCCTACCCGAAGACCAAA |
| SmHCT-Q-R | ATGGGCGTGGCTGTGAA |
| SmCHS-Q-F | CGCGATTATGCTTGAGGTTGA |
| SmCHS-Q-R | CACTACTTGATGTCCCATTTCTTGAC |
| SmDFR-Q-F | ACTGGAAGGAATGGGGTTCTC |
| SmDFR-Q-R | TTTGAGTAGAAAACGGCAGCAA |
|  |  |
| **Primers used for the amplification of JAZ fragements** | |
| SmJAZ1-F | CTCTCTGATCGGAGCAAAGATGGTT |
| SmJAZ1-R | TGATGGATCTGAAACACTAATGGCG |
| SmJAZ2-F | AGACCCCCTCTCTCTCTAGACATG |
| SmJAZ2-R | CTAAGACAGTCACAGCATCATAATTTG |
| SmJAZ3-F | ATGCAGTGGTCATTCTCCAACAAGG |
| SmJAZ3-R | GCTTCTGCTAAAACCAGCCTCTCAAT |
| SmJAZ8-F | TCACACACACACACAAACCCTAAT |
| SmJAZ8-R | TTACTGTCTGGTAATCAGGTATGGC |
| **Primers used for vector construction** | |
| SmJAZ8-F-F | GGGGTACCATGAAGCGCAACTGCAATCTG |
| SmJAZ8-F-R | CGGGATCCTTACTGTCTGGTAATCAGGTATGGC |
| CaMV35S-F | TACAAAGGCGGCAACAAACG |
| CaMV35S-R | GCAATGGAATCCGAGGAGGT |

* gene was unigene 36977, subesequently named as *SmJAZ8*

Underline indicates a restriction site.

**References**

1. Iván FA and Edward EF, *The Arabidopsis Book*. **8**. (2010).
